# Topics of Public Interest

**Published:** 1916-05

**Authors:** 


### TOPICS OF PUBLIC INTEREST.

---

**The Eight Hour Day.** Wm. Gordon Jamison, Industrial Economist, Mch. 22, holds that this is economically impossible without curtailing productiveness to a serious degree and that it is really a demand for a wage-scale which increases pay for overtime, not a literal demand for an actual 8-hour day for the purpose of increasing opportunity for self-improvement, home life, etc. The article is obviously from a partisan standpoint, but it should be seriously considered on its merits. The main issue is one which should depend largely on economic statistics and hygienic fact. We believe that there is no question that 8 hours of intense labor, either physical or mental, is the maximum of human endurance, with the ordinary allowances of a brief lunch recess, Sundays and a short annual vacation. But very few men actually work under these conditions. The experience of the medical profession, for example, and that of mankind generally, up to very recent times, indicates that most men can work a good deal more than eight hours a day without injury, according to existing standards, but it should be considered, not merely academically, but from the practical scientific standpoint, that the regular reduction of hours of labor might result in mental, physical and social improvement, increased longevity, even in economic improvement in the way of increased production, far beyond our conceptions of human possibilities. Certain manufacturers have declared that the reduction of hours of labor, without reduction in wages, has paid in increased productiveness, simply because of lessened fatigue and greater satisfaction. This matter should be studied impartially, unsentimentally, scientifically, for different kinds of work.

So far as wages are concerned, we hold that each kind of labor should receive the maximum possible, according to its actual value, except that, at the minimum of value, due regard should be given to the sociologic factors implied. In one sense, we agree with the cynical dictum that no man is worth more than a dollar a day up to his head, but it pays society to see that he does receive more, in order that his children, if not himself, shall have a head, not to mention a healthy body, that is worth more to the community than a dollar a day. The maximum possible wage can be easily determined automatically, in dealing with labor problems on a large scale. So long as the increase of wage results in corresponding greater productiveness, so long as it does not **necessarily,** result in general hardship affecting, among others, laborers of essentially the same grade and kind of work, by increasing prices of necessities unduly, so long as the recipient of the increased wage finds that it is a genuine increase and not counterbalanced by a compensatory reduction in the purchasing power of his money, wages should be increased. But we cannot get away from the fact that the real unit of value is the average day's work, not the dollar. Hence if the laborer finds that his increased wages are balanced by diminished purchasing power of the dollar, and further finds that potential employers have difficulty in getting enough money to keep him employed—there being a pretty definite amount of actual currency in the country—it is obvious that, even from the standpoint of his own interests, his wages are too high.

There is a further factor to be considered in regard to the eight-hour day, which may be expressed in the terms of a personal preference. We would rather work five days a week, approximately ten hours a day, and have a whole day off, so that the leisure could be used to get out into the country, than to work eight hours a day for six days. And we would rather work ten hours a day and six days a week and let the extra days accumulate into a vacation that would be still more available for rest and recreation. From this standpoint, even unemployment—and we understand that the average period of unemployment is about two months a year for many trades—is not a bad thing, provided the workman really gets his just wage, is provident enough to distribute it over the entire year and makes good use of his leisure. But, if the laborer prefers steady work, at eight hours a day, with his leisure evenly distributed in short daily periods which do not allow him to get far away from his home, his preference should be respected. It is scarcely necessary to add that, in no case should the hours be protracted so as to interfere with health and recuperative powers.

---

**Proposed Telephone Merger for Buffalo.** The proposition, obviously strongly favored by both companies, to merge the Federal and Bell systems is one of considerable importance to the medical profession. Theoretically, a single service is preferable and more economic. Practical experience, however, has shown that good service, reasonable rates, and wide spread use of telephones on account of these factors have been achieved only by competition. For example, both telephones for unlimited service cost $10 a year less than formerly for 500 messages on a single line. In the aggregate it has paid Buffalo to support the Federal system simply for the improvement, cheapening and extension of the Bell service secured, if the Federal had never been used at all. It has paid especially, those who have benefitted by the competition, without themselves incurring the expense of a double service. And, because so many have realized this, the benefit is about to be withdrawn. This does not by any means imply that the Federal has been useful merely as a competitor. It has introduced the vastly superior manual method of connection —although this merit is not appreciated by those who would rather spend two hours getting someone else to do something that they could themselves in an hour—and, like the old Edison exchange, it means business instead of social intercourse and is thus more promptly available. In one very important particular, two services, of any kind, even if paralleling each other, are an advantage over one, as they insure almost absolutely against the occasional interferences and failure which are inevitable in a single service. Many persons feel that the Public Service Commission will protect them against a rise in rates. This feeling is not justified by past experience as with railroads. Scarcely any firm or individual is so contented or so lacking in mental ability that he cannot put up a plausible argument that he is not getting enough for his services. The writer, who is neither a business expert nor eloquent can do so. Any large corporation can establish this contention by its ability to employ skillful pleaders. Moreover, past experience in other cities, Baltimore, for instance, shows that lack of telephone competition after it has been enjoyed, is followed by a return to the high rates of single service cities.

---

**The High Cost of Gasoline.** This matter concerns the medical profession in an economic way almost exactly comparable to the threatened increase in telephone rates. The steady increase in price of gasoline, by Standard and independent firms alike shows, first of all, that nothing is to be hoped for from the putative independence of the latter. The persistent increase in the face of popular protest and government investigation and talk of legislative control, means either what the small boy does when he puts his thumb to his nose and wiggles his fingers—and remember that the addressee is not merely the "public" but the government—or it means that the oil producers are absolutely sincere. The fact that gasoline reached its minimum for many years during the period of its maximum use, and when any shortage of supply such as is claimed could have been easily foreseen, is significant. Equally significant are the enormous dividends, in some cases stated at 100% declared. Statistics that have not been denied show that exportation has not increased but rather diminished, comparing 1915 with 1914. Semi-official government inspection is stated to show not only no failure of the supply in the ground, aside from the estimate that present sources will be exhausted in 27 years, but an actual increase in stores above ground. The prospective increase in demand is just what could have been calculated in advance from the curve of increasing automobile sales. It costs relatively less to pump and refine an additional amount than that needed for past consumption.

We do not need to save oil products for posterity. Each generation has been able to meet its own needs. A combustible engine fuel can theoretically be prepared from almost any source of carbon and hydrogen. Cellulose is abundant in quick growing shrubs and herbs, in waste of saw mills, in various forms of domestic and agricultural waste. In almost any form, it is possible to convert it into methyl alcohol; it is not asking too much of the future to make this chemic conversion economic nor to secure in some way the explosion of relatively non-volatile oil constituents. Natural gas, with or without compression beyond a degree to secure delivery, is already available for internal combustion engines, and liquid, volatile fuel can be prepared from it. This supply is of course exhaustible, but it will postpone for some years the prophecied failure of gasoline supplies. The enormous increase by ''cracking'' of the proportion of volatile material in crude mineral oil is already a practical fact, but has been, apparently to some degree controlled by the oil industries.

It has been announced that a cheap and practical apparatus has been perfected to secure electric power from the atmosphere—or ether. At any rate it is inconceivable that some method of delivering electric power for either stationary or locomotive engines cannot be achieved in the near future from the perfection of existing devices.

Very recently, we have been assured by an expert chemist that he has a substance which, added to ordinary kerosene, will enable its use in an ordinary carburetor. Still more significant is the fact that the Standard approves this discovery. But, of course, it is just as easy to raise the price of kerosene as of gasoline.

Let us not lose sight of the essential underlying principle, which applies not so much to gasoline as to other things, bread for example, which are more literally necessities of life. The problem, particular or general, will not be solved till the government recognizes the principle that taxation, not of course meaning a fair profit on any business, is strictly a function of government, not of private individuals or groups of private individuals. This principle has a corollary, the idea that it is the duty of government to limit prices of any actual economic necessity. Whether it does so through commissions—which have failed largely in the past— by establishing maximum prices which usually allow too large a margin beyond cost and fair profits, by taking certain businesses into its own hands or by other means, is not important. The general principle stated must be clearly enunciated before anything can be accomplished.

---

**Death Rates and Expectation of Life.** The Bureau of the Census states that the expectation at birth is 49.9 years for males, 53.2 for females. 4.975% of males die in the first month, 12.6% in the first year. The corresponding percentages for females are 3.894 and 10.46. Owing to the dangers of childbearing and special dangers connected with the reproductive organs, the chances of reaching 70 are almost exactly the same for the native male at the age of 42 and for the female at 22. Nearly 4/5 of violent deaths occur among males, and these form about 7% of all deaths, explaining the greater average longevity of women. Rural death rates remain somewhat higher than urban, though, we believe, the reverse is the case of N. Y. State.

---

**The N. Y. State Anti-Narcotic Law.** An attempt is being made to render this still more stringent, imposing further and still more impracticable obligations on physicians. The State Medical Society hopes to have the whole matter referred to a committee which will draft an improved law under medical advice.

---

**Russian War Losses** are said to have been 2,542,639, including killed, wounded, missing and prisoners, for the year 1915. This agrees fairly well with the estimate up to May 31, 1915, of 3,780,000.

---

**Filtration Plant for Batavia.** The State Conservation Commission has granted permission to build such a plant on the banks of Tonawanda Creek. (Note. This creek is rather highly polluted and it occurs to us that Batavia might better use wells, seek springs or even go to Lake Ontario).

---

**University of Buffalo Reunion.** About 200 alumni, including 50 from Buffalo on a special train, held a banquet in Rochester April 13.

---

**Leroy Water System.** A reservoir has been made at Union Corners, 5 miles distant, and 200,000,000 gallons impounded. Pumps have been entirely discarded and the standpipe is full to within a foot of the top.

---

**Antiseptic Eggs.** Oscar Riddle in the Pop. Science Monthly, describes a method of preserving eggs by which the hens are fed urotropin, "less than a gram a day" being given in capsule. Even this relatively large dose is said to be harmless.

---

**Wood Alcohol Death.** Relatively to the literature and legislation on the subject, deaths and blindness from wood alcohol are comparatively few. An unusual fatal case is reported from Bradford, April 15. A child drank a cupful which her father had poured out for some domestic use.

---

**Price Cutting.** The American Fair Trade League, 200 5th Avenue, N. Y., asks us to say a word against this evil. The Medical Profession, as consumers, naturally wishes the lowest price possible and, as workers, have in the majority of instances, maintained the minimum scale of prices compatible with existence. In any business, a price that does not allow fair wages and fair profits cannot be maintained. Often it signifies the deliberate assassination of a competitor and thus precedes a future and often permanent increase of prices. Thus, the ethics of price cutting depends on an absolute question of fact in each instance. Is the price fair or extortionate? Is a particular brand of goods really of a supposed quality or is it sold at a fancy price simply by an arbitrary ruling of the manufacturers, and is its alleged superiority merely one of prestige?

---

**Examination of Candidates for U. S. P. H. S.** will be held May 31 in Washington, and at a number of Marine Hospitals (selection being usually to suit the convenience of applicants). Applications should be made immediately to the Surgeon, Public Health Service, Washington.

---

**The Samuel D. Gross Prize** will be open for competition till Jan. 1, 1920. This prize awarded every 5 years, upon a subject in surgical pathology or practice, not over 150 printed octavo pages in length. Essays must be submitted typewritten, in English, and the author must agree to publish in book form. The prize is $1,500.

---

**Symposium on Quarantine in Infectious Diseases.** Addendum. Dr. Geo. L. Nicholas of the Dept. of Health, N. Y., wishes to correct the statement that the stool and urine examined for discharge from quarantine after typhoid should be taken "Not **later** than the tenth day after temperature becomes normal." Exactly the reverse should have been stated, namely that the samples should not be taken earlier than the tenth day.

---

**Importance of Good Roads to Physicians.** The American Highway Assn. of Washington, emphasizes this point, stating that most of the medical students who complete their courses, practice in the rural districts. This statement is not now quite accurate. Rather more than half of the population resides in cities, and the physicians are fewer proportionately, in the country and small towns. However, the main contention is correct, and it interests physicians without much regard to their location. For instance, a few days ago, we were summoned to a small town, some 60 miles from Buffalo. The case was urgent. If there had been good roads continuously, we could have seen the patient in three hours and could have returned by daylight. Most of the distance, there were good roads, but a road is no more passable than its softest mud puddle. We had to take a train, lose time at junctions, and the result was that we reached the patient

13 hours after the call, had to make examinations by artificial light, had a very brief sleep, arose "in the morning while it was yet night," and, at that, an office hour was encroached upon.

We stand, as a matter of military and peace preparedness for good roads connecting all towns of any considerable population. But, under existing circumstances, we feel that it is best to limit construction to brick and cement or some other impervious pavement. Macadamized state roads are, at times and places, compounds of oil or tar and flint. They are thus destructive of tires, dangerous and dirty. Their upkeep is relatively high, their life short, and they cannot be kept in good condition at all times. Thus, we feel that permanent improvement of highways should be limited to impervious pavement and that, otherwise, instead of laying short stretches with macadam, shale, sand and natural, round gravel should be used to render fairly good service in wet weather and far safer in dry weather, several times the mileage that can be macadamized for the same expense. Also, for the present, we believe that instead of attempting to distribute good roads fairly, permanent construction should be concentrated so as to render certain through routes passable for the whole distance.

This association offers 166 prizes of from $5 to $500 for photographs illustrating, positively or negatively, the value of good roads.

---

**The Passage of the Immigration Bill,** with insistance on an educational standard, is an important item of peace preparedness. Race prejudice has nothing to do with the matter. The interests of the colonial American and the man just off the ship, and expecting in good faith to become the ancestor of an American family are identical. Illiteracy is admittedly a problem, in a very practical sense, because lack of ability to acquire information is a large factor in poverty, disease and crime. The illiterate may have just as good a moral sense as the literate, but he is more likely to be led into crime through ignorance and by furnishing an easy victim, to tempt to crime on the part of others. For example, juvenile delinquency has, in many cases, been traced to the one item that shrewd children of illiterate foreign parents have consistently trained the latter to believe that the laws of the U. S. forbid the punishment of children. Of course, the educated criminal, civil or political, is more dangerous than the illiterate, but the educated criminal is an exception and can be dealt with in other ways. Moreover, we may just as well admit that our country is, on the whole, sufficiently populated. We need waste areas. An abundance of land means wealth, especially wealth of animal products, and we have already reached the point at which meat and animal foods generally are as costly as in most European countries in peace times. From one standpoint, we would like an abundance of cheap, illiterate, exploitable labor. But it requires no argument to show that from a broader standpoint, nothing can be worse for our ideals, self respect and development than depending on a constant influx of low grade labor to do our dirty work. The war is giving us an opportunity to assimilate our present population and to arrange our economies according to a normal increase of native population. It is none too early to pass laws aiming to hold immigration at something like reasonable figures.

---

**The Rockefeller Foundation** reports the expenditure of $1,342,561.11 during 1915. $570,559.71 was on account of new buildings for the Rockefeller Institute for Medical Research; $22,404.17 for what may broadly be termed medical benevolences, including eugenics, etc.; the remainder mainly for religious and educational purposes. While one should not look a gift horse in the mouth, we suggest that medical philanthropy of one kind or another should be maintained as nearly as possible at the total for the year past, and that a very worthy philanthropy would be the reduction in price of petroleum products.

---

**Exaggerated Losses in War.** According to a press clipping, said to be on the authority of the French Ministry of War, the losses of Germany alone, up to May 31, 1915, (10 months) totalled 4 millions—1,630,000 killed; 1,880,000 wounded; 490,000 prisoners. The estimate up to Nov. 22, 1915 (nearly 16 months) had shrunk to 3,700,000 of which 1/5 were estimated killed. An estimate by the British War Office, based on official lists published by the German government, up to April 10, 1916 (20 1/3 months) reduces the total to 2,730,917, of which 681,437 were killed. If the last figures are approximately correct, it will be noted that the first estimate was about 3 times exaggerated, proportionately. It is interesting to know that, at the rates observed in the U. S. the deaths for a country the size of Germany (65 million approximately) would amount to very nearly 100,000 a year for males of military age, 20-45. For the period of 20 1/3 months, they would be about 170,000. In other words, the military deaths are about 4 times the normal but, of course, they occur in only a part of the males of military age, and in a part less likely to die than the average, while the normal deaths continue in the part of the male population not in the army and, indeed, are probably slightly increased by the general stress.

However, without in any sense minimizing the horrors of war, these figures show that the popular conception of "killing off" an enemy, of depopulating a country, and of facing, after war has ended, an actual lack of men for productiveness, including procreation, is very greatly exaggerated.

**Centenarians.** Mrs. Mary Hickey of Binghamton, died on March 5, aged 108. Until the last week of her life, she occupied herself piecing quilts, without spectacles. Patrick Grogan, a soldier with Scott at the taking of Mexico City 1847, died March 9 (place not stated) aged 101. Cyril McGee died in Moira, N. Y., March 1, aged 106.

**College Merger.** Notice has already been made of the proposed merging of the Medico-Chirurgical College of Philadelphia with the University of Pennsylvania. It is now proposed to include Jefferson.

**Surgeon General Blue Asked to Resign.** Senator Works of California has introduced a resolution demanding that Dr. Blue resign the presidency of the A. M. A., on the ground that this organization is conducting a campaign for certain medical changes and that, therefore, a government official should not serve as an officer. It is proper that the organized medical profession should exert its influence in a reasonable degree to secure legislation and execution of laws for the public welfare. It is practically impossible that this influence could be exerted in a harmful way and, even if the political personnel of the government does not agree with medical opinion as expressed by this organization, it should recognize it as a valuable conservative guide. If the A. M. A., as an organization, attempted to exert an influence contrary to that of the profession itself, this fact would be apparent, and the influence would be nullified by the spontaneous protests of the profession itself. Grant that the medical profession may be somewhat biased in its opinions as to what the government should do in medical matters, it is, on the whole sincere and rather better able to form an opinion than those not qualified as physicians. The same may be said of the legal, engineering, teaching and any other profession. It may further be said that the President of the A. M. A. presides, that he has almost no influence in what its enemies may term the politics of the A. M. A.—even less than the influence of a majority of the fellows and members, and decidedly less than any individual who chooses to take the time and spend the money to be a "kicker." The election of Dr. Blue to the presidency of the A. M. A. was not only an honor to and appreciation of an individual physician. It was also a deference to the U. S. Government as personified by the head of one of its great medical services. The personal honor can in no way be affected by the forced resignation of Dr. Blue, who can, at most, miss its public expression by appearing before an audience, and who would be spared several days of lame wrist and crushed hand, following the annual reception. On the other hand, the somewhat ungracious refusal of a token of respect paid by the medical profession to the Government itself, especially at this late day, seems exceedingly unwise.

---

**The Bureau of Laboratories of the Buffalo Dept. of Health** will occupy the premises 484 William Street, formerly used for Police precinct No. 8. This bureau is a unification of the work in bacteriology, pathology, milk analysis, clinical diagnosis, etc., and will be under the supervision of Major Wm. G. Bissell, M. D. Two expert chemists have been added to the staff, Roy Futzer and Warren Gabriel. The building has been renovated in its interior and equipped so that this Bureau will be equal to any similar municipal enterprise in the country.

---

**The Alumni of the University of Buffalo** held a banquet in Elmira March 26. About 60 were present, including representatives of the Faculties from Buffalo.

---

**Abortion Case.** The daily press report a charge of criminal abortion against a prominent physician in Western N. Y. In accordance with our policy, we decline to give further publicity to the matter till the trial has decided the guilt or innocence of the accused.

---

**Transfer of authority at Saratoga Springs.** The legislature has passed the bill transferring the work of the Saratoga Springs Reservation Commission to the State Conservation Commissioner, Mr. George D. Pratt, of Brooklyn. The bill has gone to the Governor for signature. It is to be hoped that Mr. Pratt will appoint a deputy to represent him at Saratoga Springs who will be a good business man and able to further the development of the spa on the lines adopted by European experts.

---

**Ascaris in Tonsil.** A. B. Middleton, Clinical Jour., reports the case of an anaemic girl of eight with enlarged tonsils and adenoids. The right tonsil ruptured when the snare was tightened and a female ascaris, 1¼ inches long escaped. It had been in a cavity in the tonsil, possibly for years.

